# Virulence and Evolution of West Nile Virus, Australia, 1960–2012

**DOI:** 10.3201/eid2208.151719

**Published:** 2016-08

**Authors:** Natalie A. Prow, Judith H. Edmonds, David T. Williams, Yin X. Setoh, Helle Bielefeldt-Ohmann, Willy W. Suen, Jody Hobson-Peters, Andrew F. van den Hurk, Alyssa T. Pyke, Sonja Hall-Mendelin, Judith A. Northill, Cheryl A. Johansen, David Warrilow, Jianning Wang, Peter D. Kirkland, Stephen Doggett, Christy C. Andrade, Aaron C. Brault, Alexander A. Khromykh, Roy A. Hall

**Affiliations:** The University of Queensland, St. Lucia, Queensland, Australia (N.A. Prow, J.H. Edmonds, Y.X. Setoh, H. Bielefeldt-Ohmann, W.W. Suen, J. Hobson-Peters, A.A. Khromykh, R.A. Hall);; CSIRO Australian Animal Health Laboratory, Geelong, Victoria, Australia (D.T. Williams, J. Wang);; The University of Queensland, Gatton, Queensland, Australia (H. Bielefeldt-Ohmann, W.W. Suen);; Department of Health, Brisbane, Queensland, Australia (A.F. van den Hurk, A.T. Pyke, S. Hall-Mendelin, J.A. Northill, D. Warrilow);; The University of Western Australia, Nedlands, Western Australia, Australia (C.A. Johansen);; Elizabeth Macarthur Agriculture Institute, Menangle, New South Wales, Australia (P.D. Kirkland);; University of Sydney and Pathology West–ICPMR, Westmead, New South Wales, Australia (S. Doggett);; University of California, Davis, California, USA (C.C. Andrade, A.C. Brault);; Centers for Disease Control and Prevention, Fort Collins, Colorado, USA (C.C. Andrade, A.C. Brault)

**Keywords:** West Nile virus, virulence, evolutionary markers, viruses, Australia, encephalitis, zoonoses, flavivirus, vector-borne infections

## Abstract

Worldwide, West Nile virus (WNV) causes encephalitis in humans, horses, and birds. The Kunjin strain of WNV (WNV_KUN_) is endemic to northern Australia, but infections are usually asymptomatic. In 2011, an unprecedented outbreak of equine encephalitis occurred in southeastern Australia; most of the ≈900 reported cases were attributed to a newly emerged WNV_KUN_ strain. To investigate the origins of this virus, we performed genetic analysis and in vitro and in vivo studies of 13 WNV_KUN_ isolates collected from different regions of Australia during 1960–2012. Although no disease was recorded for 1984, 2000, or 2012, isolates collected during those years (from Victoria, Queensland, and New South Wales, respectively) exhibited levels of virulence in mice similar to that of the 2011 outbreak strain. Thus, virulent strains of WNV_KUN_ have circulated in Australia for >30 years, and the first extensive outbreak of equine disease in Australia probably resulted from a combination of specific ecologic and epidemiologic conditions.

## Abstract 2

Despite the absence of disease in humans and animals, virulent virus strains have been circulating for >30 years.

West Nile virus (WNV) is a mosquito-transmitted flavivirus that causes encephalitis. Outbreaks of potentially fatal neurologic syndromes have occurred in Europe and Africa (*1*); recently, however, strains of WNV have caused large outbreaks of encephalitis in humans and horses in the Americas and Australia (*2*,*3*). The Kunjin strain of WNV (WNV_KUN_) is indigenous to Australia and historically has caused only relatively mild, nonfatal disease in humans and horses. However, in 2011, a large unprecedented outbreak of encephalitis in horses, involving ≈900 reported cases, occurred in southeastern Australia; a high proportion of cases were attributed to the emergence of a virulent strain of WNV_KUN_ (*3*,*4*). WNV_KUN_ has been shown to be enzootic to northern Australia and to have episodic activity in southern regions thought to be associated with periods of heavy rainfall (*5*). However, the epidemiology of WNV_KUN_ seems to have changed over the past decade; virus activity has been detected in the absence of prior flooding and in areas where it was previously not detected (*4*).

Studies comparing the virulence of various WNV strains in mouse models have identified several motifs, residing in both structural and nonstructural genes as well as in the 5′ and 3′ untranslated regions (UTRs). These motifs were associated with enhanced viral invasion of the central nervous system and onset of neurologic disease (*5*–*11*). 

To identify potential markers of virulence of WNV_KUN_ in Australia, we investigated evolutionary mechanisms behind the emergence of virulent strain(s) by using established mouse models to compare the neuroinvasive properties of WNV_KUN_ isolates collected from different regions of Australia during 1960–2012. To investigate known markers of WNV virulence, we conducted comparative analyses of viral genome sequences. 

## Methods

### Cell Culture, Virus Production, and Titration

We used 13 WNV_KUN_ strains isolated during 1960–2012 (Table 1) and African green monkey kidney (Vero) and *Aedes albopictus* mosquito (C6/36) cells, cultured as previously described (*3*). The 1960 prototype WNV_KUN_ strain (WNV_KUN1960_) was used as the attenuated reference virus and was either an unknown passage of the original isolate (WNV_MRM16_) or derived from an infectious clone of a plaque-purified virus (WNV_MRM61C_) (*12*,*13*). These isolates were previously shown to be phenotypically identical (*13*,*14*). WNV strain NY99-4132 was obtained from the US Centers for Disease Control and Prevention (Fort Collins, CO, USA) and used as a virulent control. Virus stocks and methods for determination of infectious titers have been described (*3*).

**Table 1:** WNV_KUN_ strains used during study of virulence and evolution of WNV, Australia, 1960–2012*

Isolate	Year collected	Location	Source	Passage history
MRM16/MRM61C†	1960	Mitchell River Mission, Queensland	Mosquito‡	Unknown
Boort	1984	Victoria	Horse spinal cord	Unknown
K2499	1984	Kimberley region, Western Australia	Mosquito‡	2× C6/36; 1× PSEK
Hu6774	1991	New South Wales	Human	Unknown
K6453	1991	Kimberley region, Western Australia	Mosquito‡	2× C6/36; 1× PSEK
SH183	1991	Victoria	Chicken	Unknown
Gu0631	2000	Gulf of Carpentaria, Queensland	Mosquito‡	3× C6/36
Gu1009	2000	Gulf of Carpentaria, Queensland	Mosquito‡	3× C6/36
K68967	2009	Kimberley region, Western Australia	Mosquito‡	3× C6/36
P9974	2009	Pilbara region, Western Australia	Mosquito‡	3× C6/36
NSW2011	2011	New South Wales	Horse brain	2× C6/36; 1 Vero
K74015	2011	Kimberley region, Western Australia	Mosquito‡	3× C6/36
NSW2012	2012	New South Wales	Mosquito‡	3× C6/36

*C6/36, from *Aedes albopictus* mosquitoes; PSEK, porcine squamous equine kidney cells; WNV, West Nile virus; WNV_KUN_, Kunjin strain of WNV. †Prototype strain. ‡*Culex annulirostris.*

### Antigenic Analysis

We compared reactivity of WNV_KUN_ isolates with a panel of monoclonal antibodies (mAbs) with that of reference strains WNV_KUN1960_ and WNV_NY99._ To do so, we used a fixed-cell ELISA, as previously described (*3*,*5*,*15*).

### Virus Replication Kinetics

We performed growth kinetics analysis by infecting Vero and C6/36 cells at a multiplicity of infection of 1 at 37°C (Vero) or 28°C (C6/36) with WNV_KUN_. Culture supernatants were harvested at 0, 24, 48, and 72 h after infection (Vero) and 0, 24, 48, 72, 96, and 120 h after infection (C6/36) and titrated (*3*). Statistical significance from 3 independent experiments was determined by using 2-way analysis of variance following log transformation (*16*). Mean virus titers were compared between viruses by using the Tukey method for pairwise multiple comparisons (GraphPad Prism, version 6.0; GraphPad Software Inc., San Diego, CA, USA).

### Virulence in Mice

Performance of all animal procedures was approved by The University of Queensland Animal Ethics Committee. To determine virus virulence in mice, we intraperitoneally inoculated 20 Swiss white outbred CD-1 mice (weanlings [18–19 days of age] and young adults [28 days of age]) with a range of doses (0.1–10,000 PFU) of each WNV strain (Table 1) (*3*). The significance of clinical differences between groups was calculated by Kaplan-Meier analysis and analyzed by log-rank test where noted (GraphPad Prism, version 6.0). A virus strain was designated as virulent if survival times for mice infected with this strain (both age groups) differed significantly from those of mice infected with the attenuated reference strain WNV_KUN1960_. All virus strains that did not meet this criterion for virulence were designated as attenuated.

### Full-length Genome Sequencing

We sequenced 9 WNV_KUN_ genomes (Technical Appendix Table 1) by using random primer sequencing on extracts of C6/36 cell cultures (*17*). Viral RNA was extracted by using a MagMAX-96 Viral RNA Isolation Kit (Ambion, Waltham, MA, USA) according to the manufacturer’s instructions. cDNA synthesis and random PCR amplification were conducted according to previously described methods (*18*), and resultant PCR amplicons were used for sequencing library preparation. DNA libraries were prepared by using a Nextera XT DNA Sample Preparation Kit (Illumina, San Diego, CA, USA) according to the manufacturer’s protocols. Paired-end sequencing of 150-bp fragments was performed by using a MiSeq Reagent Kit V2 (300 cycles) and MiSeq Sequencing System (Illumina). Sequencing data were analyzed by using CLC Genomics Workbench version 6.5.0 (http://www.clcbio.com**).** The sequence data were trimmed by using quality scores specified in CLC Genomics Workbench before performing read-mapping analysis. The genome sequences were assembled by read mapping against the reference WNV_KUN_ strain genome (GenBank accession no. JX276662) with use of default parameters in the mapping algorithm. Where gaps in the genome sequence or low coverage (<10 reads/site) were observed, conventional Sanger sequencing was performed to complete or verify the sequence. Oligonucleotide primers sequences designed for these purposes are available upon request to A.A.K or R.A.H.

### Bioinformatics Analysis

We used MUSCLE, as implemented in MEGA6 (*19*), to align complete open reading frame (ORF) (10,320 nt) and partial (402 nt) envelope (E) gene nucleotide sequences of the newly sequenced WNV_KUN_ strains, together with those available for 6 other WNV_KUN_ strains and selected WNV isolates, representing different lineages. We estimated maximum-likelihood phylogenetic trees by using PhyML version 3.0 (*20*) and by using substitution models and rates among sites selected with JModelTest version 2.1.5 (*21*). We tested reliability of the inferred trees by using the bootstrap method with 1,000 replicates. All trees were rooted with analogous ORF sequences from Murray Valley encephalitis virus (GenBank accession no. NC000943) and Japanese encephalitis virus (GenBank accession no. EF571853) and visualized by using FigTree version 1.4.0 (http://tree.bio.ed.ac.uk/software/figtree/). Pairwise distances were determined at the nucleotide and amino acid levels by using the p-distance model in MEGA6.

## Results

### WNV_KUN_ Strains

A panel of previously characterized mAbs (*5*,*15*,*22*–*24*) was used to antigenically type 13 WNV_KUN_ strains. The binding profiles of these mAbs confirmed that all WNV_KUN_ isolates closely resembled the prototype WNV_KUN1960_ strain, including strong recognition by mAb 10A1, known to be specific for WNV_KUN_ strains (Table 2) (*2*,*24*). Only WNV_KUN_ strains isolated before 2000 were recognized by mAb 5H1, which binds a linear epitope in the αA3 motif (residues 39–53) in the methyltransferase domain of nonstructural (NS) protein 5 (*15*). Lack of 5H1 binding was associated with a substitution at residue 49 (Ile-Val) in αA3 (Table 3); this finding was consistent with previous mutagenesis study findings that a substitution of Ile to Ala at this residue was associated with abolition of 5H1 binding (*26*).

**Table 2:** Binding patterns of monoclonal antibodies to WNV strains in ELISA*

Strain	Year of isolation	Monoclonal antibodies, by specificity							
		Panflavivirus, 4G2, anti-env	Pan-WNV, 2B2, anti-env	WNV_KUN-_specific			Glycosylated E, 17D7	Unglycosylated E, 3.101C	MVEV-specific, 10C6
				10A1, anti-env	5D4, anti-NS5	5H1, anti-NS5			
WNV_KUN_†									
KUN1960	1960	+	+	+	+	+	–	+	–
Boort	1984	+	+	+	+	+	+	–	–
K2499	1984	+	+	+	+	+	+	–	–
SH183	1991	+	+	+	+	+	+	–	–
K6453	1991	+	+	+	+	+	+	–	–
Hu6774	1991	+	+	+	+	+	+	–	–
Gu0631	2000	+	+	+	+	+	+	–	–
Gu1009	2000	+	+	+	+	+	+	–	–
K68967	2009	+	+	+	+	–	+	–	–
P9974	2009	+	+	+	+	–	+	–	–
NSW2011	2011	+	+	+	+	–	+	–	–
K74015	2011	+	+	+	+	–	+	–	–
NSW2012	2012	+	+	+	+	–	+	–	–
Reference									
WNV_NY99_	1999	+	+	–	+	–	+	–	–
MVEV_1–51_	1951	+	–	–	–	–	–	–	+

*Binding of a monoclonal antibody was designated as positive if the optical density was at least double the optical density of the negative control (mock-infected C6/36 cells). E, envelope protein; MVEV, Murray Valley encephalitis virus; NS5, nonstructural protein 5; WNV, West Nile virus; WNV_KUN_, Kunjin strain of WNV; +, positive; –, negative.  †WNV_KUN_ strains collected during 1960–2012, Australia.

**Table 3:** Amino acid sequences in the West Nile virus genome*

WNV strain	Year of isolation	prM, residue 22/72†	Putative virulence determinant			NS5, residue 49	3′ UTR residues 64–71
			E protein, residues 154–156‡	NS3, residue 249§	NS5, residue 653¶		
NY99	1999	Val/Ser	Asn-Tyr-Ser (NYS)	Pro	Phe	Val	Present
KUN1960	1960	Ile/Leu	Asn-Tyr-Phe (NYF)	Ala	Ser	Ile	Present
Boort	1984	Ile/Leu	Asn-Tyr-Ser (NYS)	Ala	Phe	Ile	Present
K2499	1984	Ile/Leu	Asn-Tyr-Ser (NYS)	Ala	Phe	Ile	Present
K6453	1991	Ile/Leu	Asn-Tyr-Ser (NYS)	Ala	Phe	Ile	Present
Hu6774	1991	Ile/Leu	Asn-Tyr-Ser (NYS)	Ala	Phe	Ile	Present
Gu0631	2000	Ile/Leu	Asn-Tyr-Ser (NYS)	Ala	Phe	Ile	Present
Gu1009	2000	Ile/Leu	Asn-Tyr-Ser (NYS)	Ala	Phe	Ile	Absent
K68967	2009	Ile/Leu	Asn-Tyr-Ser (NYS)	Ala	Phe	Val	Absent
P9974	2009	Ile/Leu	Asn-Tyr-Ser (NYS)	Ala	Phe	Val	Absent
NSW2011	2011	Ile/Leu	Asn-Tyr-Ser (NYS)	Ala	Phe	Val	Absent
K74015	2011	Ile/Leu	Asn-Tyr-Ser (NYS)	Ala	Phe	Val	Absent
NSW2012	2012	Ile/Leu	Asn-Tyr-Ser (NYS)	Ala	Phe	Val	Absent

*E, envelope; NS, nonstructural; prM, premembrane; UTR, untranslated region.  †(*11*). ‡(*7*). §(*25*). ¶(*10*).

### Glycosylated E Proteins

All analyzed WNV_KUN_ isolates collected after 1960 contain glycosylated E proteins. The sequence analysis of the E gene of WNV_KUN_ isolates revealed the presence of a conserved potential N-linked glycosylation site at residue 154 in all but the prototype WNV_KUN1960_ isolate (Table 3). To confirm that this site was indeed glycosylated on the viral E protein, each virus was assessed for recognition in ELISA by mAbs 17D7 and 3.101C (Table 2), which specifically recognize glycosylated and unglycosylated WNV_KUN_ E proteins, respectively (*5*,*23*). The results supported the predictions from our sequencing data; all WNV_KUN_ strains except the prototype WNV_KUN1960_ strain were recognized by mAb 17D7 but not by 3.101C.

### Growth Kinetics and Plaque Morphology of WNV_KUN_ Strains

**Figure 1:**
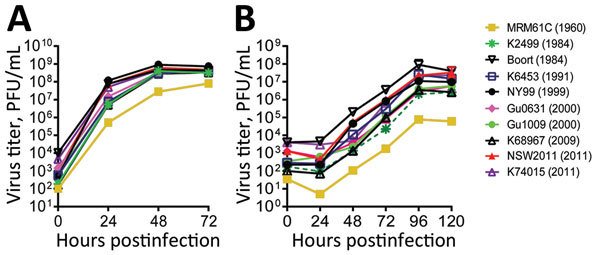
Growth kinetics of West Nile virus strains isolated in Australia, 1960–2012, in Vero (A) and C6/36 (B) cells. Cells were infected with a multiplicity of infection of 1, and the virus titers in the supernatants were determined by plaque assay on Vero cells.

Infection of Vero cells at 24 h postinfection demonstrated that WNV_KUN1960_, WNV_K2499_, WNV_K6453_, and WNV_K68967_ isolates yielded significantly lower titers than did WNV_NY99_ (p<0.05) (Figure 1, panel A). However, by 48 h postinfection, similar titers were reached for all WNV isolates except WNV_KUN1960_.

A similar trend was observed in C6/36 cells, in which viral titers of WNV_KUN1960_, WNV_K2499_, WNV_K68967_, and WNV_GU1009_ were significantly lower than those for WNV_NY99_, WNV_NSW2011_, and WNV_Boort_ at 48 h after infection (p<0.05) and titers for WNV_K6453_, WNV_K74015_, and WNV_GU0631_ were intermediate. By 96 h after infection, the titers of all WNV isolates except WNV_KUN1960_ were similar.

**Figure 2:**
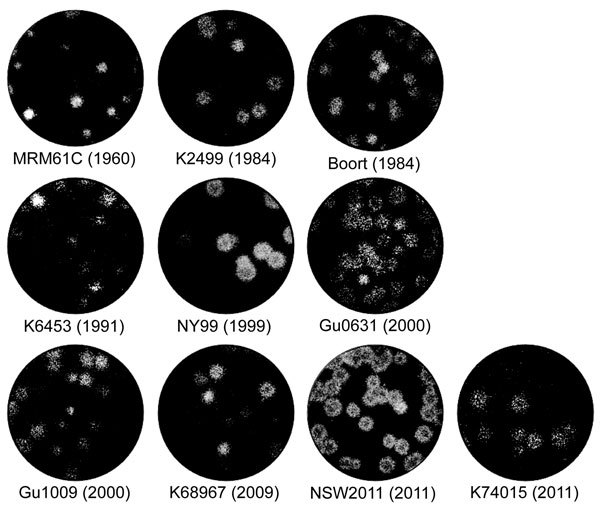
Plaque morphology of representative West Nile virus strains isolated in Australia, 1960–2012. Virus was allowed to adsorb to monolayers of Vero cells for 2 h at 37**°**C. The cells were then overlaid with Dulbecco Modified Eagle Medium containing 0.5% low melting point agarose and 2% fetal bovine serum. Four days after infection, the cells were fixed with 4% formaldehyde solution and stained with 0.2% crystal violet.

In terms of plaque morphology of WNV_KUN_ strains, in Vero cells, WNV_NSW2011_ and WNV_K74015_ produced large plaques (average size 4.3 ± 0.63 and 4.3 ± 0.77 mm, respectively), a size similar to those produced by the WNV_NY99_ strain (average size 4.8 ± 0.45 mm). The prototype virus, WNV_KUN1960_, produced very small plaques (average size 2.7 ± 0.47 mm), which differed significantly from those of all other viruses tested during this study (p<0.0001). The remaining isolates produced intermediate-sized plaques (average size 3.5–3.9 ± 0.45–0.84 mm) (Figure 2) (*5*). Plaques formed by WNV_K6453_, WNV_K74015_, WNV_Gu1009_, and WNV_Gu0631_ were less well defined than those formed by WNV_NY99_, WNV_NSW2011_, WNV_K2499_, and WNV_Boort_.

### Virulence of WNV_KUN_ Strains in Mice

**Figure 3:**
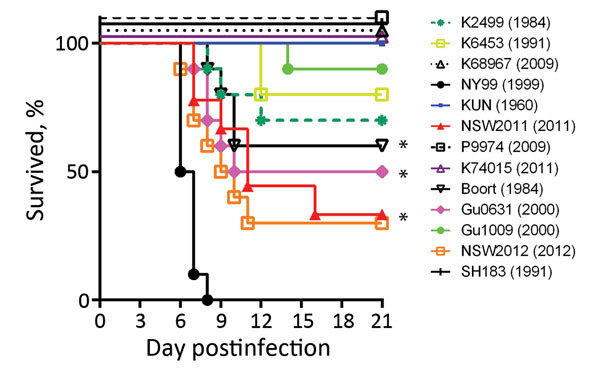
Survival curves for young adult (28-day-old) Swiss outbred mice after intraperitoneal infection with 1,000 PFU of West Nile virus (WNV) strains isolated in Australia, 1960–2012. Groups of 10 mice were infected with each virus. The mice were monitored for 21 days after infection for signs of encephalitis and then euthanized. WNV_NY99_ and WNV_NSW2011_ with previously demonstrated virulence were included as controls. The significance of clinical differences between groups was calculated by Kaplan-Meier analysis and analyzed by log-rank test. Significantly increased virulence over that of WNV_KUN1960_ is indicated by an asterisk (*): WNV_Boort_ (p = 0.0295), WNV_Gu0631_ (p = 0.0115), and WNV_NSW2012_ (p = 0.0011). No significant differences were observed between WNV_Boort_, WNV_Gu0631_, and WNV_NSW2012_ compared with WNV_NSW2011_ (p>0.05).

We previously demonstrated that differentiation between virulent and attenuated strains of WNV can be detected in weanling and young adult mice (*3*,*27*). In this study, we found that in addition to WNV_NSW2011_, 3 other WNV_KUN_ isolates (WNV_Boort_, WNV_Gu0631_, WNV_NSW2012_) were neuroinvasive in both mouse models (Figure 3; Technical Appendix Table 2). The WNV_Boort_ strain, obtained from the spinal cord of a symptomatic horse during a small outbreak of equine disease in southeastern Australia in 1984, was neuroinvasive in young adult mice (40% mortality rate at 1,000 PFU); this finding did not statistically differ in this respect from that for WNV_NSW2011_ (p = 0.3218) (Figure 3). Two other isolates obtained from mosquitoes, 1 from the Gulf of Carpentaria in 2000 (WNV_Gu0631_) and 1 from southeastern Australia in 2012 (WNV_NSW2012_), also exhibited levels of neuroinvasive properties in young adult mice similar to those caused by WNV_NSW2011_ (Figure 3). In weanling mice, the virulence of WNV_Boort_, WNV_Gu0631_, and WNV_NSW2012_ was also similar to that of WNV_NSW2011_; mortality rates, 50% lethal dose, or time to death did not differ significantly (Table 4). Of note, WNV_Gu1009_ isolated at the same time and from the same region as WNV_GU0631_ was significantly less virulent in young adult mice (Figure 3; Technical Appendix Table 2). The remaining isolates were relatively attenuated in both young adult (Figure 3) and weanling (Technical Appendix Table 2) mice and did not differ significantly from the attenuated prototype WNV _KUN1960_ strain (p>0.05).

**Table 4:** Comparison of amino acid sequences between virulent and attenuated West Nile virus strains*

Gene and amino acid position in polyprotein (corresponding protein)	NY99	Virulent strains		Attenuated Kunjin strains						
		Boort, NSW2011, NSW2012, Gu0631		KUN1960	K68967	K2499	K6453	K74015	Hu6774	Gu1009
C										
86 (86)	Lys	Lys		Arg	Lys	Lys	Lys	Lys	Lys	Lys
114 (114)	Met	Ile (Boort), Thr (NSW2011, NSW2012, Gu0631)		Met	Thr	Thr	Thr	Met	Thr	Thr
prM										
143 (20)	Thr	Ala		Thr	Ala	Ala	Ala	Ala	Ala	Ala
158 (35)	Ile	Thr		Ile	Thr	Thr	Thr	Thr	Thr	Thr
279 (156)	Val	Thr		Ala	Thr	Thr	Thr	Thr	Thr	Thr
E										
413 (123)	Thr	Thr		Ala	Thr	Thr	Thr	Thr	Thr	Thr
446 (156)	Ser	Ser		Phe	Ser	Ser	Ser	Ser	Ser	Ser
600 (310)	Lys	Arg		Thr	Arg	Arg	Arg	Arg	Arg	Arg
773 (483)	Leu	Phe		Leu	Phe	Phe	Phe	Leu	Phe	Phe
790 (500)	His	His		Tyr	His	His	His	His	His	His
NS1										
1081 (290)	Ser	Asn		Ser	Asn	Asn	Asn	Ser	Asn	Asn
NS2A										
1255 (112)	Val	Val		Ala	Val	Val	Val	Val	Val	Val
1272 (129)	Ile	Ile		Met	Ile	Ile	Ile	Ile	Ile	Ile
1366 (223)	Ile	Ile		Val	Ile	Ile	Ile	Ile	Ile	Ile
NS3										
1520 (146)	Lys	Lys		Arg	Lys	Lys	Lys	Lys	Lys	Lys
1970 (586)	Asn	Ser		Asn	Ser	Ser	Ser	Asn	Ser	Ser
NS4A										
2179 (55)	Ala	Thr		Ala	Thr	Thr	Thr	Thr	Thr	Ala
NS4B										
2296 (23)	Val	Ile		Thr	Ile	Ile	Ile	Ile	Ile	Ile
2324 (51)	Val	Phe		Val	Phe	Phe	Phe	Val	Phe	Phe
2368 (95)	Ala	Ser		Ala	Ser	Ser	Ser	Ser	Ser	Ser
2450 (177)	Met	Ile		Met	Ile	Ile	Ile	Ile	Ile	Ile
2518 (245)	Ile	Ile		Val	Ile	Ile	Ile	Ile	Ile	Ile
NS5										
2629 (101)	Arg	Lys		Arg	Lys	Lys	Lys	Arg	Lys	Lys
3088 (560)	Asp	Asn		Asp	Asn	Asn	Asn	Asp	Asn	Asn

*C, capsid; E, envelope; NS, nonstructural; prM, premembrane.

### Sequence of Viral Genomes

**Figure 4:**
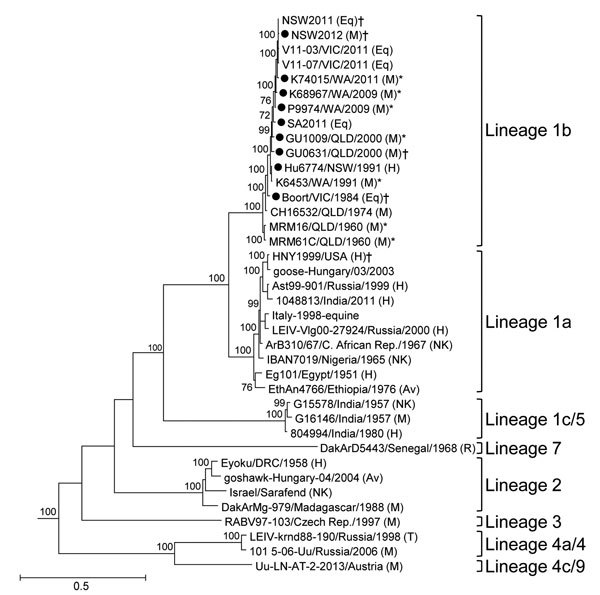
Maximum-likelihood phylogenetic tree estimated by using nucleotide sequences of the complete open reading frame (ORF) of genomes of West Nile virus (WNV) strains isolated in Australia, 1960–2012 (black circles), compared with representative strains from different lineages and clades. The tree was estimated by using a general time-reversible model of nucleotide substitution with a gamma distribution and invariant sites. Bootstrap values are shown on the nodes and are expressed as a percentage of 1,000 replicates; only values >70% are shown. Horizontal branch lengths indicate genetic distance. The tree was rooted with the ORF sequences of Murray Valley encephalitis virus and Japanese encephalitis virus; however, these branches have been removed to improve resolution. Strains that were assessed as having an attenuated virulence phenotype are indicated by a single asterisk (*), and virulent strains are indicated by a dagger (†).The state of origin for WNV_KUN_ strains is shown as follows: NSW, New South Wales; QLD, Queensland; SA, South Australia; VIC, Victoria; WA, Western Australia. Virus sources are indicated in parentheses next to virus identity, as follows: Av, avian; Eq, equine; H, human; M, mosquito; NK, not known; R, rodent; T, tick. Scale bar indicates nucleotide substitutions per site.

We sequenced WNV_KUN_ isolates to analyze their relationship to the prototype WNV_KUN_ strains from 1960 (WNV_MRM16_, WNV_MRM61C_); the 2011 outbreak strains (WNV_NSW2011_, WNV_SA2011_, WNV_V11-03_, and WNV_V11-07_); and exotic strains of WNV known to be virulent in humans and horses (WNV_NY99_) or representing different WNV lineages. Phylogenetic analysis of the ORF sequences demonstrated that the WNV_KUN_ strains form a single genetically homogeneous clade within lineage 1 (Figure 4), as previously recognized (*24*); nucleotide and amino acid identities between strains were 96.1%–99.4% and 98.5%–100%, respectively. As expected, the most recent isolates, including the 2011 outbreak strain, were the most divergent, and the early prototype strains (WNV_MRM61C_ and WNV_MRM16_) occupied the basal lineage of this clade. Recent strains isolated in 2011 and 2012 from southeastern Australian states clustered together and shared high levels of nucleotide (98.6%–100%) and amino acid (99.6%–100%) identities, indicating transmission of a genetically homogeneous virus population during this period. These strains were either virulent for horses or shown in this study to be virulent in mice (Figure 3; Technical Appendix Table 2). No other association between phylogenetic relationships and virulence was found; other virulent strains clustered closely and interspersed with the attenuated strains identified in this study. An expanded phylogenetic analysis that used 45 partial E gene sequences (402 nt) and a larger range of reference WNV_KUN_ strains showed a similar pattern of relationships (Technical Appendix Figure).

Of note is the very close relationship (99.9% aa identity) between a virulent 2011 strain isolated from a horse and an isolate obtained from *Culex annulirostris* mosquitoes trapped in New South Wales, Australia, in 2012 (WNV_NSW2012_). Only 3 nonconservative changes were identified between WNV_NSW2011_ and WNV_NSW2012_, located in NS1 (Lys33Arg), NS3 (Phe509Leu), and NS4A (Phe92Leu). These results suggest that the virulent strain either had persisted in New South Wales after the end of the 2011 outbreak or had been reintroduced to the area.

Analyses of predicted gene products from the complete ORF sequence of each WNV_KUN_ isolate revealed that, in addition to the glycosylation site at residues 154–156 in the E protein, all strains isolated after 1960 contained a Phe residue at position 653 in the NS5 protein, which has previously been shown to play a role in resistance to antiviral activity of interferon-α/β (*10*) (Table 3). In contrast, WNV_KUN1960_ contained a Ser residue at position 653 in NS5 (*3*,*5*,*24*,*28*). The Pro residue at position 249 in the NS3 protein, previously shown to be present in WNV strains and associated with increased virulence in birds of some species (*25*), was not present in any of the WNV_KUN_ isolates, which all contained an Ala residue at this position (Table 3).

In addition to an Ile→Val substitution at position 49 in NS5 of WNV_KUN_ isolates collected after 2009, analysis of more contemporary WNV_KUN_ isolates also revealed a consistent 8-nt deletion in the 3′ UTR, just downstream from the ORF stop codon. This deletion was identified in WNV_Gu1009_ and all isolates collected after 2000. In contrast, this deletion was not present in isolates collected before 2000 (Table 3) or in another isolate from Gulf of Carpentaria collected in 2000 (WNV_Gu0631_). We suggest that these 2 features (Ile→Val 49 residue in NS5 and an 8-nt deletion in the 3′ UTR) can be considered as potential evolutionary markers.

In addition to the genetic variability described above, sequence analysis between virulent and attenuated WNV_KUN_ strains identified other nucleotide differences between isolates, located throughout the viral genome. These differences result in amino acid substitutions (Table 4) and may contribute to observed phenotypic differences.

We also sequenced WNV_KUN_ viral RNA extracted directly from mosquito saliva expectorated onto sugar-soaked nucleic acid preservation cards placed in mosquito traps in Darwin, Northern Territory, in 2012 (WNV_NT2012_) (*29*,*30*). When partial sequences from E, NS5, and the 3′ UTR from this RNA were aligned, we observed a high level (99.7%) of identity with the WNV_KUN1960_ strains, indicating that viruses genetically homologous to the prototype virus are still circulating in some regions of Australia (online Technical Appendix Figure). Closer analysis revealed a lack of E glycosylation, similar to that found in the prototype strain. However, Phe was identified at position 653 of NS5, similar to that found in recent isolates.

## Discussion

Historically, WNV_KUN_ has been associated with only mild disease in humans and rare cases of disease in horses, consistent with data from mouse virulence studies that revealed a relatively attenuated phenotype (*3*,*8*,*27*). Thus, the emergence of an equid-virulent strain of WNV_KUN_, responsible for ≈900 cases of encephalitis in horses in southeastern Australia, was unprecedented. 

Although most WNV_KUN_ isolates examined in this study exhibited an attenuated phenotype, similar to that of the prototype WNV_KUN1960_, we identified an additional 3 strains with neuroinvasive properties in mice similar to those reported for WNV_NSW2011_ (*3*). The first, WNV_Boort_, was isolated from the spinal cord of a horse with nonsuppurative encephalomyelitis and severe neurologic symptoms in northern Victoria in 1984 (*31*). At that time, 53 animals in the same area were clinically affected. However, a high incidence of Ross River virus–specific antibody in these animals implicated that virus rather than WNV_KUN_ as the primary etiologic agent (*31*). Our results are also supported by another recent study showing virulence of WNV_Boort_ in 18–19-day-old mice (*32*).

The second virulent strain identified in this study, WNV_Gu0631_, was isolated from *Cx. annulirostris* mosquitoes collected from Normanton, Gulf of Carpentaria, in April 2000. Of note, this virus was isolated in the absence of any reported disease outbreak, as part of a survey for the presence of Japanese encephalitis virus in northern Queensland (*33*). The second Gulf of Carpentaria isolate, WNV_Gu1009_, was also collected in April 2000, from the town of Karumba, which is ≈30 km from Normanton. However, WNV_GU1009_ is genetically distinct and attenuated to the same degree as the prototype WNV_KUN1960_ in 28-day-old mice (Figure 4). These observations demonstrated that virulent WNV_KUN_ strains might co-circulate with attenuated strains in some regions of Australia. Furthermore, the circulation of neuroinvasive strains may often appear in the absence of disease outbreaks. This suggestion is consistent with our finding that WNV_NSW2012_ was genetically almost identical to the WNV_NSW2011_ and exhibited similar levels of neuroinvasiveness in mice. However, no cases of disease in equids were associated with WNV_KUN_ infection during the 2012 season (*3*,*4*,*34*). This lack of cases suggests that the persistence of virulent strains in southeastern Australia is not the sole determinant for initiating disease outbreaks and that specific climatic and ecologic conditions, perhaps influencing mosquito populations and viral transmission, are also required.

A similar scenario occurred in North America, where an unusually high number of cases in humans (5,387), most in Texas, USA, were reported in 2012. However, sequence analysis of WNV isolates from 2012 revealed that the strains circulating in Texas were virulent and attenuated, and no specific virulence determinants responsible for the increase in cases could be identified (*35*). Instead, other factors, including temperature and changes in mosquito or bird populations, were speculated to have contributed to the magnitude of the 2012 outbreak (*36*).

To identify a phylogenetic association with virulence and to identify potential virulence determinants encoded in the genome of WNV_KUN_ strains, we also performed full-length sequencing of the ORF of several of the viruses studied. Although recent virulent strains were phylogenetically closely related, no other association between phylogenetic grouping and virulence phenotype was found (Figure 4; Technical Appendix Figure). One notable change in the genome that was clearly associated with the temporal distribution of these viruses was a highly conserved 8-base deletion in the 3′ UTR, just downstream of the ORF stop codon. Isolates from samples collected after 2000, including the virulent WNV_NSW2011_ and attenuated strains, invariably contained this deletion. This finding suggests that the deletion is an evolutionary marker but is not directly associated with virulence. This finding is also consistent with our observation that the neuroinvasive 2000 Gulf of Carpentaria isolate, WNV_Gu0631_, did not have this deletion but that the co-circulating attenuated isolate, WNV_Gu1009_, collected from the same region at the same time, did have this deletion.

An additional evolutionary change was observed in the α-A3 motif of the methyltransferase domain of the NS5 protein. Isolates obtained before 2009, including the prototype WNV_KUN1960_, contained a conserved Ile residue at position 49. However, all isolates collected after 2009 displayed an Ile→Val substitution at this position. Coincidentally, this substitution abolished the binding of a mAb (5H1) that recognizes a linear epitope comprising the α-A3 peptide (*15*).

Initial comparisons between the virulent isolate WNV_NSW2011_ from a horse and the attenuated prototype WNV_KUN1960_ revealed that several previously identified WNV virulence markers were detected in the former but not in the latter isolate (*3*). These markers included the conserved N-linked glycosylation of the E protein (*7*) and the Phe residue at position 653 in the NS5 protein, associated with resistance to antiviral activity of interferon α/β (*8*). Although these initial observations suggested the involvement of these motifs in the enhanced neuroinvasive properties of the isolate collected from a horse in 2011, our study revealed that, with the exception of WNV_KUN1960_, all strains examined contain both of these markers, regardless of virulence phenotype in mouse models. Thus, it seems that, although these motifs contribute to virulence in mice, they are not likely to be solely responsible for enhancing the neuroinvasive properties of some WNV_KUN_ strains and, hence, not likely to be markers of evolving virulence in recent isolates of WNV_KUN_.

Additional markers of WNV virulence identified in WNV strains from North America were not detected in any of the WNV_KUN_ isolates. This finding is consistent with our repeated observations that even the equid-virulent WNV_NSW2011_ is substantially less neuroinvasive than WNV_NY99_ in young adult mice (*3*). These motifs may include Val 22 and Ser 72 residues in the premembrane, which enhance mouse neuroinvasiveness when introduced into the prototype WNV_KUN1960_ (*11*), and the Pro residue at position 249 in NS3, which is associated with enhanced virulence in birds (*25*). The absence of the latter motif in all WNV_KUN_ strains is also consistent with the perceived lack of illness and death among birds in Australia, notably during the 2011 outbreak among equids. Some isolates included in this study (including WNV_KUN1960_, WNV_SH183_, WNV_Boort_, and WNV_Hu6774_) have an unknown passage history. Extensive passage through cells is known to occasionally lead to passage-adapted mutations, and care should be taken when interpreting sequencing data from these virus strains.

WNV_KUN_ is thought to be endemic to the tropical areas of northern Australia, suggesting that virulent viruses emerging in southeastern Australia probably originate from northern Australia. However, WNV_KUN_ recently isolated from mosquitoes in northern Australia, including the 2011 Kimberley isolate WNV_K74015_, were more attenuated than WNV_NSW2011_. This finding suggests a different explanation for the evolution of virulent WNV_KUN_ viruses, which may be associated with the adaption of WNV_KUN_ to different hosts (avian and terrestrial) or different vector species in temperate regions. In this context, virulence in equids may be just a coincidental outcome of the constraints placed on virus fitness in different geographic locations (*35*–*37*). 

Overall, our results show that virulent strains of WNV_KUN_ have been circulating in Australia for >30 years and that the first extensive outbreak of disease among horses in Australia in 2011 probably resulted from a combination of ecologic and epidemiologic conditions rather than the emergence of a novel, more virulent strain. Further studies evaluating viral fitness of West Nile virus quasispecies in terms of population-dependent host–virus interactions, are warranted.

Technical Appendix GenBank accession numbers and details of West Nile virus (WNV) strains used for phylogenetic analysis; virulence of WNV strains in 18–19-day-old mice; maximum-likelihood phylogenetic tree of WNV Kunjin and reference strains.
