# Supplementary material for: Virulence and Evolution of West Nile Virus, Australia, 1960–2012
Source: Emerg Infect Dis. 2016 Aug;22(8):1353–62. doi: 10.3201/eid2208.151719 (PMC4982165; doi:10.3201/eid2208.151719)
Supplement: Technical Appendix — GenBank accession numbers and details of West Nile virus (WNV) strains used for phylogenetic analysis; virulence of WNV strains in 18–19-day-old mice; maximum-likelihood phylogenetic tree of WNV Kunjin and reference strains. [file 15-1719-Techapp-s1.pdf]

Article DOI: http://dx.doi.org/10.3201/eid2208.151719

Virulence and Evolution of West Nile Virus, Australia

Technical Appendix

Technical Appendix Table 1. GenBank accession numbers and details of WNV strains used for phylogenetic analysis in study of virulence and evoloution of WNV, Australia

Isolate	Location	Year collected	Source	GenBank accession no.
MRM16	Mitchell River Mission, QLD	1960	*Cx. annulirostris*	GQ851602
MRM61C	Mitchell River Mission, QLD	1960	*Cx. annulirostris*	D00246
MRM5373	Mitchell River Mission, QLD	1966	*Cx. annulirostris*	AF196509
OR134	Kimberley region, WA	1973	*Cx. annulirostris*	AF196506
OR4	Kimberley region, WA	1973	*Cx. annulirostris*	AF196523
OR205	Kimberley region, WA	1973	*Cx. annulirostris*	AF196515
OR130	Kimberley region, WA	1973	*Cx. annulirostris*	AF196492
OR166	Kimberley region, WA	1973	*Cx. annulirostris*	AF196499
CH16532C	Charleville, QLD	1974	*Cx. annulirostris*	JX276662
CH16549E	Charleville, QLD	1974	*Cx. annulirostris*	AF196520
CH16514C	Charleville, QLD	1974	*Cx. annulirostris*	AF196501
CH16465C	Charleville, QLD	1974	*Cx. annulirostris*	AF196504
OR393	Kimberley region, WA	1974	*Cx. annulirostris*	AF196503
OR354	Kimberley region, WA	1974	*Cx. annulirostris*	AF196518
WK436	Kimberley region, WA	1979	*Cx. annulirostris*	AF196507
CX238	Kimberley region, WA	1982	*Cx. annulirostris*	AF196502
M695	VIC	1982	*Cx. annulirostris*	AF196496
M1465	VIC	1983	*Cx. annulirostris*	AF196522
V407	Jabiru, NT	1983	*Cx. annulirostris*	AF196508
Boort*	Boort, VIC	1984	Horse spinal cord	KT934796
K2499	Kimberley region, WA	1984	*Cx. annulirostris*	AF196498
FC15	Kimberley region, WA	1986	*Cx. annulirostris*	AF196510
K5374	Kimberley region, WA	1989	*Cx. annulirostris*	AF196517
K1738	Kimberley region, WA	1989	*Cx. annulirostris*	AF196494
Hu6774*	Southern NSW	1991	Human	KT934797
K6453	Kimberley region, WA	1991	*Cx. annulirostris*	GQ851603
K6590	Kimberley region, WA	1991	*Cx. annulirostris*	AF196500
K6547	Kimberley region, WA	1991	*Cx. annulirostris*	AF196521
SH183	VIC	1991	Chicken	AF196491
P1553	Kimberley region, WA	1994	*Cx. species*	AF196495
CY3404	Cape York, QLD	2000	*Cx. sitiens* subgroup	AY251614
Gu0631*	Gulf of Carpentaria, QLD	2000	*Cx. annulirostris*	KT934798
Gu1009*	Gulf of Carpentaria, QLD	2000	*Cx. annulirostris*	KT934799
TS5656	Torres Strait, QLD	2000	*Cx. sitiens* subgroup	AY251611
TS5677	Torres Strait, QLD	2000	*Cx. sitiens* subgroup	AY251612
TS5750	Torres Strait, QLD	2000	*Cx. sitiens* subgroup	AY251613
TS5926	Torres Strait, QLD	2000	*Cx. sitiens* subgroup	AY251610
K68967*	Kimberley region, WA	2009	*Cx. annulirostris*	KT934802
P9974*	Pilbara region, WA	2009	*Cx. annulirostris*	KT934800
NSW2011	Eastern NSW	2011	Horse brain	JN887352
SA2011*	Hallett, SA	2011	Horse brain	KT934803
K74015*	Kimberley region, WA	2011	*Cx. annulirostris*	KT934801
V11-03	VIC	2011	Horse CNS tissue	JX123030
V11-07	Euroa, VIC	2011	Horse CNS tissue	JX123031
NSW2012*	Eastern NSW	2012	*Cx. annulirostris*	Submitted (1863956)
NT S3083	Leanyer swamp, NT	2012	Mosquito (FTA card)	KC492441
Eg101	Cairo, Egypt	1950	Human	AF404756
EthAn4766	Ethiopia	1976	Bird	AY603654
Italy-1998-equine	Tuscany, Italy	1998	Horse	AF404757
LEIV-Vlg00-27924	Volgograd, Russia	2000	Human	AY278442
Ast99-901	Volga delta, Russia	1999	Human	AY603654

Isolate	Location	Year collected	Source	GenBank accession no.
1048813	Kerala, India	2011	Human	KC601756
HNY1999	New York, USA	1999	Human	AF202541
goose-Hungary/03	Hungary	2003	Goose	DQ118127
IBAN7019	Nigeria	1965	Unknown	GQ851607
ArB310/67	Central African Republic	1967	Unknown	GQ851608
Sarafend	Israel	Unknown	Unknown	AY688948
Eyoku	Democratic Republic of Congo	1958	Human	HM147824
DakArMg-979	Madagaskar	1988	*Cx. quinquefasciatus*	HM147823
goshawk-Hungary-04	Hungary	2004	Goshawk	DQ116961
Rabensburg (97-103)	South Moravia, Czech Republic	1997	*Cx. pipiens*	AY765264
LEIV-krnd88	NW Caucasus, Russia	1998	*Dermacentor marginatus*	AY277251
101_5-06-Uu	Volgograd, Russia	2006	*Uranotaenia unguiculata*	FJ159129
G16146	India	1957	Mosquito	KJ831223
G15578	India	1957	Not known	GQ851604
804994	Bangalore, India	1980	Human	DQ256376
DakArD5443	Senegal	1968	*Tatera kempi* (rodent)	EU082200
WNV-Uu-LN-AT-2013	Burgenland State, Austria	2013	*Ur. unguiculata*	KJ831223

*WNV_{KUN} strains sequenced as part of this study.

Technical Appendix Table 2. Virulence of West Nile virus strains in 18–19-d–old mice after intraperitoneal infection

Virus strain and dose, pfu	No. mice died/no. mice injected	Average survival time, d	LD_{50}
NY99*			
100	10/10	6.1	
10	10/10	6.7	
1	10/10	6.9	0.1 pfu
0.1	5/10	7.8	
KUN (1960)[#*]			
1000	9/10	8.4	
100	4/10	8	
10	6/10	10.2	13.4 pfu
1	3/10	12	
NSW2011*			
1000	10/10	7.1	
100	10/10	7.4	
10	10/10	7.7	
1	7/10	8.3	0.5 pfu
0.1	1/10	10	
NSW2012			
10	10/10	7.4	
1	7/10	8.3	0.5 pfu
0.1	1/10	10	
K2499			
1000	10/10	7.4	
100	10/10	7.8	
10	9/10	9.5	2.0 pfu
1	3/10	17.1	
0.1	0/12	21	
K6453			
1000	6/10	12.4	
100	5/10	15.4	
10	4/10	17.1	
1	1/10	20.1	100 pfu
0.1	0/10	21	
K68967			
1000	10/10	7.4	
100	10/10	7.8	
10	5/10	14.3	10 pfu
1	4/10	15.7	
0.1	0/10	21	
K74015			
10	5/10	9	10 pfu
P9974			
1000	9/10	8.5	
100	9/10	10.3	

Virus strain and dose, pfu	No. mice died/no. mice injected	Average survival time, d	LD_{50}
10	5/10	15.5	10 pfu
1	1/10	19.9	
0.1	0/10	21	
SH183			
100	10/10	7	
10	8/10	10.1	2.5 pfu
1	3/10	17.7	
Boort			
100	10/10	6.9	
10	10/10	7.2	
1	9/10	9.4	
0.1	5/10	15.1	0.1 pfu
Gu1009			
100	9/10	9.3	
10	9/10	10.1	
1	9/10	10	0.4 pfu
0.1	2/10	18.9	
Gu0631			
100	10/10	7.2	
10	10/10	7.6	
1	10/10	7.8	0.2 pfu
0.1	2/10	18.6	

*Reference virus, data from (*1*).

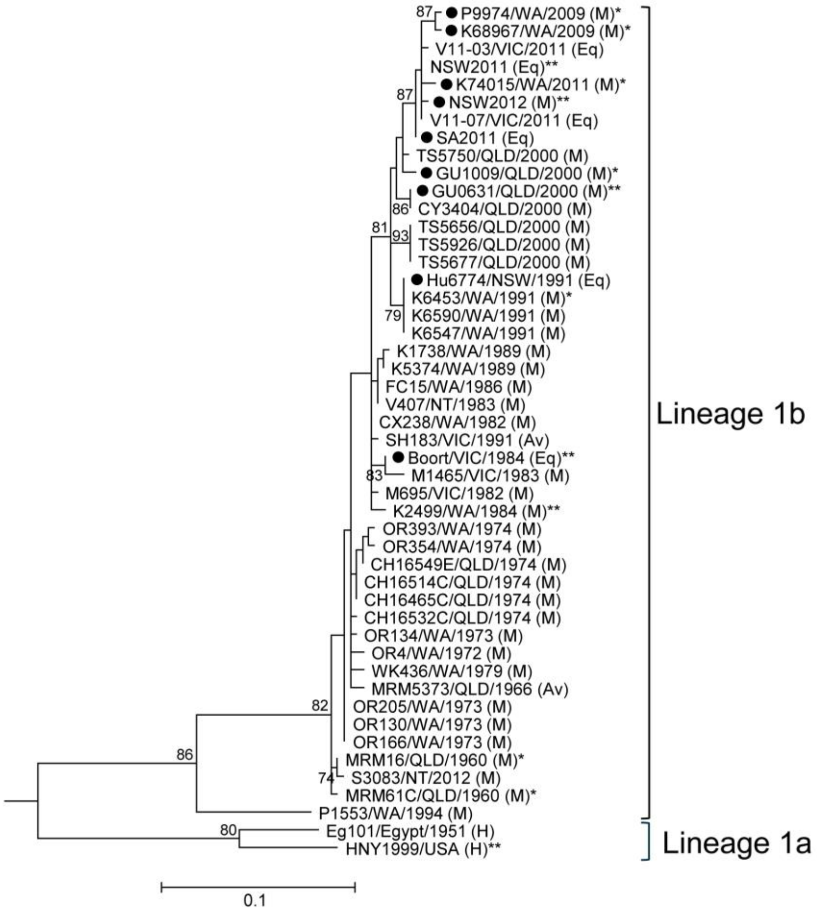

Technical Appendix Figure. Maximum-likelihood phylogenetic tree estimated using partial envelope gene sequences (402 nt) of WNV_{KUN} strains and reference WNV strains. The tree was estimated using a general time-reversible model of nucleotide substitution with a gamma distribution and invariant sites. Bootstrap values are shown on the nodes and are expressed as a percentage of 1,000 replicates; values >70% only are shown. Horizontal branch lengths indicate genetic distance proportional to the scale bar. The tree generated using the same reference sequences as for the open reading frame phylogeny and was rooted with Murray Valley encephalitis and Japanese encephalitis sequences. To improve resolution, all branches of reference sequences have been removed except for lineage 1, clade a. Strains sequenced as part of this study are indicated by a closed circle. Those that were assessed as having an attenuated virulence phenotype are indicated by a single asterisk, while virulent strains are indicated by a double asterisk. The state of origin for WNV_{KUN} strains is shown with the following abbreviations: NSW, New South Wales; QLD, Queensland; SA, South Australia; VIC, Victoria; WA, Western Australia. Virus source is indicated in parentheses next to its identity, as follows: Av, avian; Eq, equine; H, human; M, mosquito. WNV, West Nile virus.

Reference

1. Frost MJ, Zhang J, Edmonds JH, Prow NA, Gu X, Davis R, et al. Characterization of virulent West Nile virus Kunjin strain, Australia, 2011. Emerg Infect Dis. 2012;18:792–800. PubMed http://dx.doi.org/10.3201/eid1805.111720
